# GaPO_4_ Single Crystals: Growth Condition by Hydrothermal Refluxing Method

**DOI:** 10.3390/molecules25194518

**Published:** 2020-10-02

**Authors:** Denis Balitsky, Etienne Philippot, Vladimir Balitsky, Ludmila Balitskaya, Tatiana Setkova, Tatiana Bublikova, Philippe Papet

**Affiliations:** 1 Cristal Laser SAS, 54850 Messein, France; 2 ICGM, CNRS-UM-ENSCM, UMR 5253, Université Montpellier, Place Eugène Bataillon, CEDEX 5, 34095 Montpellier, France; philippot.etienne@orange.fr (E.P.); philippe.papet@umontpellier.fr (P.P.); 3 D.S. Korzhinskii Institute of Experimental Mineralogy of Russian Academy of Sciences (IEM RAS), 142432 Chernogolovka, Russia; balvlad@iem.ac.ru (V.B.); lvbalitskaya@mail.ru (L.B.); setkova@iem.ac.ru (T.S.); tmb@iem.ac.ru (T.B.)

**Keywords:** piezoelectric crystals, gallium orthophosphate, crystal growth, hydrothermal method

## Abstract

Bulk GaPO_4_ is an advanced piezoelectric material operating under high temperatures according to the α-β phase transition at 970 °C. This work presents the technological development of a hydrothermal refluxing method first applied for GaPO_4_ single crystal growth. Crystals of 10–20 g were grown in mixtures of aqueous solutions of low- and high-vapor-pressure acids (H_3_PO_4_/HCl) at 180–240 °C (10–20 bars). The principal feature of the refluxing method is a spatial separation of crystal growth and nutrient dissolution zones. This leads to a constant mass transportation of the dissolved nutrient, even for materials with retrograde solubility. Mass transport is carried out by dissolution of GaPO_4_ nutrient in a dropping flow of condensed low-vapor-pressure solvent. This method allows an exact saturation at temperature of equilibrium and avoids spontaneous crystallization as well loss of seeds. Grown crystals have a moderate OH^−^ content and reasonable structural uniformity. Moreover, the hydrothermal refluxing method allows a fine defining of GaPO_4_ concentration in aqueous solutions of H_3_PO_4_, H_2_SO_4_, HCl and their mixtures at set T–P parameters (T < 250 °C, *p* = 10–30 bars). The proposed method is relatively simple to use, highly reproducible for crystal growth of GaPO_4_ and probably could applied to other compounds with retrograde solubility.

## 1. Introduction

The development of modern radio communication devices and different kinds of electronic measurement technologies puts increasing demands on the requirements of piezoelectric inventory materials. Generally, this means the values of the electromechanical constants and its temperature dependencies. The most widely used quartz crystals have an α–β phase transformation at 573 °C [1]. Thus, application of quartz piezoelectric devices is limited to approximately 300–350 °C [2]. There have been many studies investigating new alpha-quartz-like structure piezoelectric materials with homogeneous compositions, low dielectric losses even at high temperatures and significant piezoelectric properties in a wide temperature range exceeding 350 °C [3].

The best piezoelectric coefficients of α-quartz-like structure materials have been found for α-GeO_2_. This compound is stable in the α-quartz structural phase in a temperature range of 1033 to 1080 °C. It has a metastable α-quartz structure state at temperatures up to 180 °C for crystals grown by hydrothermal method [4,5] and much higher for flux-grown crystals [3,6]. Currently, the use of the flux method (top-seeded solution growth (TSSG)) allows crystallization on a seed up to 100 g, but the crystals contain many defects [7].

Another known high-temperature α-quartz-like structure material is GaPO_4_. The temperature of α-quartz structural stability is nearly 930 °C [8,9]. Its piezoelectric coefficients are slightly lower than for α-GeO_2,_ but the quality and volume of crystals crystallized by the proposed refluxing hydrothermal method look both more suitable and reproducible than GaPO_4_ single crystals [10,11].

The existing methods of GaPO_4_ crystal growth [12,13,14,15,16,17,18,19,20,21] are hydrothermal methods, retrograde temperature gradient “slow temperature rise” up to 250 °C methods, direct temperature gradient (above 320 °C) methods based on retrograde (at T < 250 °C) methods and direct (at T > 320 °C) fields of GaPO_4_ solubility in aqueous solutions, accordingly. A possibility of GaPO_4_ crystallization under high-temperature conditions in molybdates fluxes has also been confirmed [22]. All these methods allow growing GaPO_4_ crystals, but with a series of restrictions. The crystals grown at temperatures up to 250 °C in aqueous H_3_PO_4_ or H_3_PO_4_/H_2_SO_4_ solutions have a considerable OH^−^ content. Crystals grown in aqueous H_2_SO_4_ solution have a low OH^−^ content, but growth rates of {0001} faces equal to zero that leads to formation of plate-like crystals. Additionally, the use of hydrothermal methods up to 250 °C is associated with a number of technique-engineering complexities such as a preliminary saturation of crystal growth solutions and inconstant temperature of equilibrium. Crystal growth by the direct temperature gradient hydrothermal method at 320 °C or higher needs very complex equipment (Pt lined high-pressure autoclaves with an inert gas-injection system) [12,13,17]. The GaPO_4_ crystals grown from fluxes are very small (millimeters size) and contains impurities coming from the flux [22,23,24,25,26,27,28].

**Figure 1:**
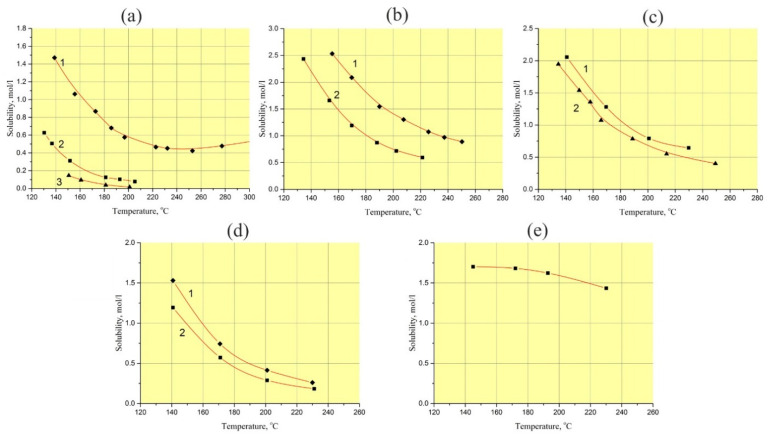
Solubility of GaPO_4_ in aqueous solutions (at pressure of 5–20 bar): (**a**) H_3_PO_4_, 1–15 *M*, 2–7.5 *M*, 3–5 *M*; (**b**) H_2_SO_4_, 1–9 *M*, 2– 6.5 *M*; (**c**) 15 *M* H_3_PO_4_/9 *M* H_2_SO_4_ in vol. proportions (%): 1–20/80, 2–50/50; (**d**) 9 *M* H_3_PO_4_/6 *M* H_2_SO_4_ in vol. proportions (%): 1–20/80, 2–70/30; (**e**) 6 *M* HCl [30].

Using the retrograde temperature gradient hydrothermal method at temperatures up to 250 °C, it is impossible or very difficult to maintain a given supersaturation of the solution all times of the run and as a result, reproducibility of such experiments has a low level. The slow temperature rise hydrothermal method is limited by GaPO_4_ solubility [8,29]. The constant value of GaPO_4_ solubility in aqueous acid solutions (H_3_PO_4_, H_2_SO_4_, HCl and their mixtures) in the temperature range from 200 to 260 °C (Figure 1) completely excludes using of this method at temperatures higher than 180 °C [30].

A detail study of GaPO_4_ crystal growth conditions has revealed some optimal parameters for obtaining crystals with a low OH- content and reasonable structural homogeneity (low crack density and gas–liquid impurities). The papers [10,18,29,31] describe that low OH^−^ content in GaPO_4_ crystals was fixed with the following parameters: temperature higher than 220 °C, solvent of aqueous H_3_PO_4_, H_2_SO_4_ or a mixture of these solutions. Unfortunately, growing bulk GaPO_4_ crystals by known methods at such temperatures is difficult or impossible. The principal problem facing GaPO_4_ crystal growth at temperatures up to 230 °C is the low convection of the solution, which obstructs mass transportation. The practically constant solubility of GaPO_4_ in acids at temperatures of 200–260 °C (see Figure 1) [30] is the reason of unsuccessful crystal growth. Nevertheless, it looks more promising to use a hydrothermal method combining, if possible, relatively high growth rates, low density of defects, low OH^−^ content and reproducible volumetric crystallization.

Analyzing existing hydrothermal methods of the crystal growth, we have found a hydrothermal refluxing method. In particular, this method was applied to α-GeO_2_ single-crystal growth [11,32]. It is characterized by high and stable convection of the solvent during crystal growth cycle and a controlled solubility of nutrients in a quantity necessary for the continuous crystal growth.

## 2. Results

### 2.1. Study of GaPO_4_ Concentration in the Solutions

**Figure 2:**
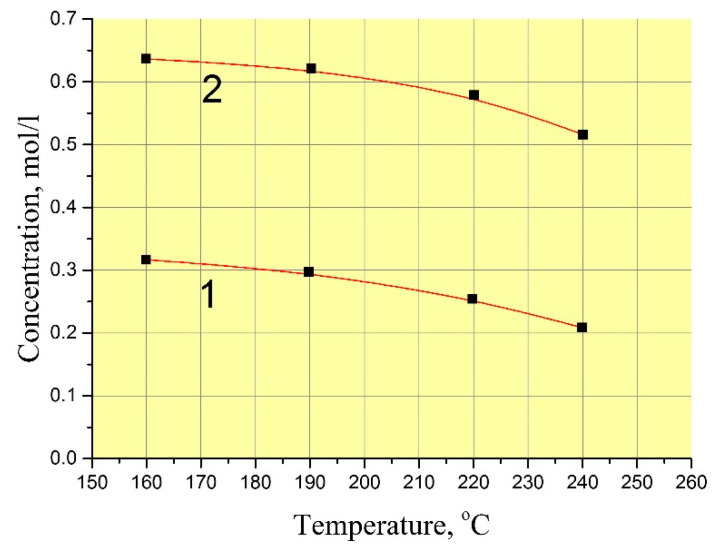
GaPO_4_ concentration in 9 *M* H_3_PO_4_/ 6 *M* HCl (vol. proportion 95/5%) at temperature 160–240 °C and pressure 5–20 bar. 1—with crystallization; 2—without crystallization.

**Figure 3:**
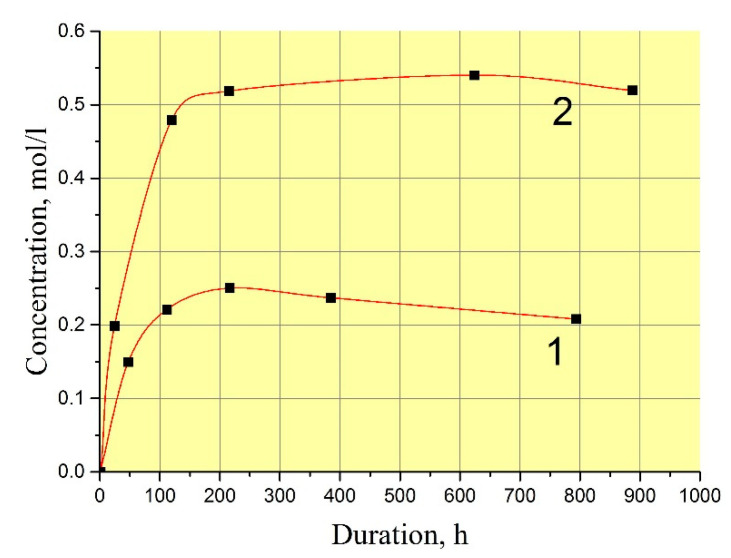
Kinetics of GaPO_4_ dissolution in 9 *M* H_3_PO_4_/6 *M* HCl (vol. proportion 95/5%) at 240 °C and 10–15 bar. 1—with crystallization; 2—without crystallization.

The exact values of GaPO_4_ solubility are not very important for the refluxing method in terms of reproducing of crystal growth at different temperatures. In practice, the procedure of the crystal growth run described before leads to reproducible restart conditions without any undesirable spontaneous crystallization or seed loss. However, scientific interest have moved us to measure GaPO_4_ concentration in the solutions. The low filling of autoclave by solution (f < 60%) in the refluxing method allowed us to use a technique of autoclave reversing to stop the dissolution at a set temperature. This enabled us to keep the nutrient into the initial solution during the run and to stop dissolution cycle at a set temperature by reversing the autoclave. Two values of GaPO_4_ concentration were found in the mixing 9 *M* H_3_PO_4_/6 *M* HCl (vol. proportion 95/5%) at temperatures 160–240 °C: with and without crystallization at the same T–P conditions (Figure 2) as well the kinetics of saturation (Figure 3). Likely, this indicates a quite large zone of metastability of GaPO_4_ crystallization. The measured kinetics of saturation is relatively slow: more than 100 h before it becomes constant. This could be explained by a low convection or stationary conditions in the solution.

The concentration of GaPO_4_ in 9 *M* H_3_PO_4_/12 *M* HCl solution (vol. proportion– 95/5%) showed a large difference between the values without and with crystallization (see Figure 1). This suggests that the starting conditions of GaPO_4_ crystal growth are very “soft”. Formation of different kinds of defects on the surface of seeds in a very beginning of crystallization are low or not desirable under such conditions.

The influence of the low-vapor-pressure solvent additive, presented by HCl, changed the character of GaPO_4_ solubility compared to pure aqueous H_3_PO_4_, H_2_SO_4_ or their mixtures. The concentration of GaPO_4_ in solutions used for the refluxing method is a constant at temperatures of 150–250 °C and pressures up to 10–20 bar.

### 2.2. Main Results of the Crystal Growth

More than 20 runs with durations up to 35 days of GaPO_4_ crystal growth were completed. Crystals were grown with weights up to 20 g. After each run, the growth rates of crystals and concentration of GaPO_4_ were measured. The crystal habit and OH^−^ content of crystals (by IR spectroscopy) were determined. The main results of the runs are presented in Table 1.

**Figure 4:**
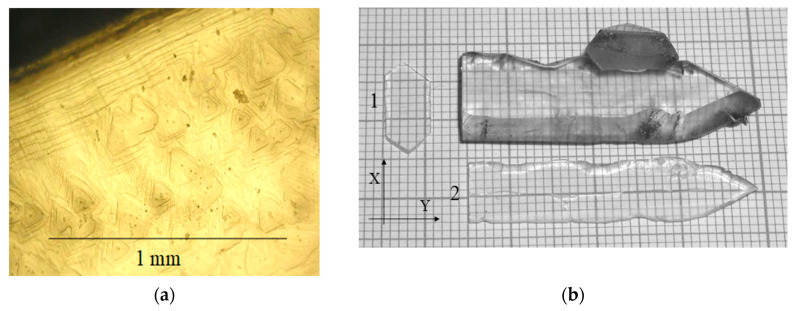
(**a**) Face of basal pinacoid c{0001} is represented by layer-dislocation growth mechanism, at gradients up to 15 °C; (**b**) GaPO_4_ crystal grown on ZY seed plate and plates cut from this crystal: 1—cut perpendicular to Y axis, 2—perpendicular to Z axis.

**Figure 5:**
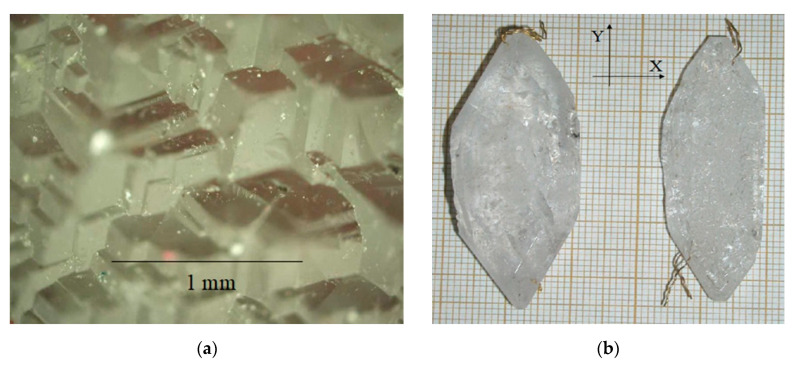
(**a**) Poly-head face of basal pinacoid c{0001}, formed by trigonal rhombohedral pyramids π’{$01\bar{1}2$} or z{$01\bar{1}1$}, gradients above 15–20 °C; (**b**) GaPO_4_ crystal grown on ZY seed plate.

The face of basal pinacoid c{0001} is flat at temperature gradients up to 15 °C, it is represented by layer (Figure 4a). At gradients above 15–20 °C, the basal face is a poly-head constructed by trigonal pyramids π’{$01\bar{1}2$} or z{$01\bar{1}1$} (Figure 5a). Growth rates of basal pinacoids were different for the mentioned cases: at low gradients, it was up to 0.2 mm/day for two opposite faces; at high gradients—more than 0.4 mm/day for two opposite faces.

The internal structure of basal pinacoid growth sectors was more homogenous in the first case under conditions of low temperature gradients (Figure 4b). At growth rates of 0.4 mm/day and more, the gas–liquid inclusions and multiple dislocations were formed (Figure 5b).

**Figure 6:**
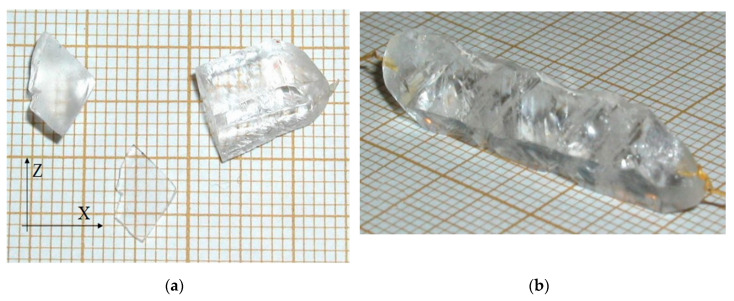
GaPO_4_ crystals grown on x{$12\bar{1}0$} seeds, (**a**) plate cut perpendicular to Y axis, (**b**) typical habit of the crystals represented by appearance of the trigonal rhombohedral faces interrupting formation of negative and positive trigonal prism faces.

Crystal growth on trigonal prism faces was characterized by high velocities, more than 1 mm/day. However, the crystals grown on x{$12\bar{1}0$} seeds were small because of trigonal pyramid faces appearance interrupting formation of negative and positive trigonal prism faces (Figure 6a,b).

**Figure 7:**
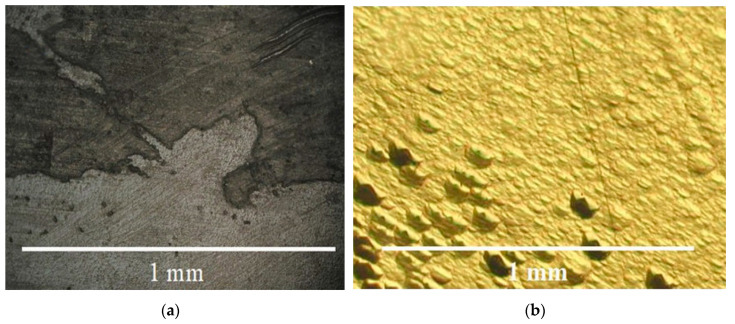
(**a**) Stairs and (**b**) low hillocks of the rhombohedral faces < z > and < π’ >.

**Figure 8:**
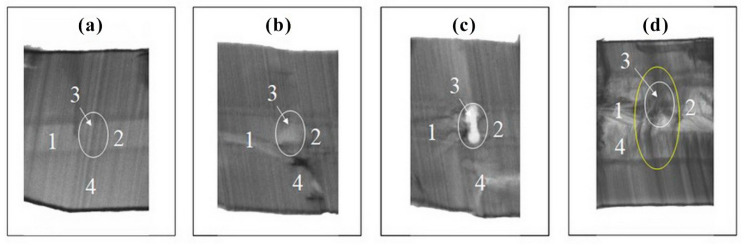
X-ray topography of cross sections of GaPO_4_ crystals grown on c {0001} seed plates (thickness is near to 1.5 mm) included some defects: (**a**) twin, (**b**) dislocation, (**c**) crack and (**d**) all together. 1, 2—initial seed plates; 3—defect (twin, dislocation or crack), 4—overgrown layer.

Crystal growth of all trigonal rhombohedron and trigonal dipyramid faces was presented by layer or dislocation mechanism. Stairs (Figure 7a) and low hillocks (Figure 7b) were present on the rhombohedron faces. Propagation of Brazil twins from seed was observed in sectors of the rhombohedron faces (Figure 8a–d). Any gas–liquid inclusions, dislocations and cracks (see Figure 8b–d) were not typical for sectors of the rhombohedron face growth of GaPO_4_ crystals.

### 2.3. Growth Kinetics

Growing of GaPO_4_ crystals by the refluxing method in aqueous solution of H_3_PO_4_–HCl at temperatures of 200–240 °C and pressures up to 30 bar showed a strong influence of temperature gradient to the crystal habit. However, the growth rates sequence of different faces of the crystals was constant. The following sequence of growth rates took place for GaPO_4_ crystals obtained by the refluxing method:

x{$12\bar{1}0$} > s{$11\bar{2}\;0$} > r{$10\bar{1}1$} > c{0001} > π{$10\bar{1}2$} > z{$01\bar{1}1$} > π’{$01\bar{1}2$}

This growth rate sequence was in a good correlation with similar ones of GaPO_4_ crystals grown from aqueous solutions of H_2_SO_4_ by the temperature gradient method at temperatures of 170–240 °C and pressures of 10–30 bar.

### 2.4. IR Spectroscopy

The influence of OH^−^ content on electromechanical properties of piezoelectric crystals is well known [3,33]. It is more suitable to call it a hydrogen impurity which is frequently occurs forming a bound to an oxygen and as a result forms the hydroxyl (OH^−^). This could be in different forms: independent OH^−^ bonds—in unstructural impurities and OH^−^ bonds in substitution to cations—as interstitial defects. Regardless, the resulting OH^−^ bond is highly polar and as a consequence this dipole absorbs the infrared radiation.

It was found that the wave numbers near to 3400, 3290 and 3167 cm^−1^ of such absorption are the vibrations of OH^−^ bonds of GaPO_4_ crystals [34,35,36]. For comparing the observed data to other works [18,22,31,34,35,36] we used only the wave number at 3400 cm^−1^.

**Figure 9:**
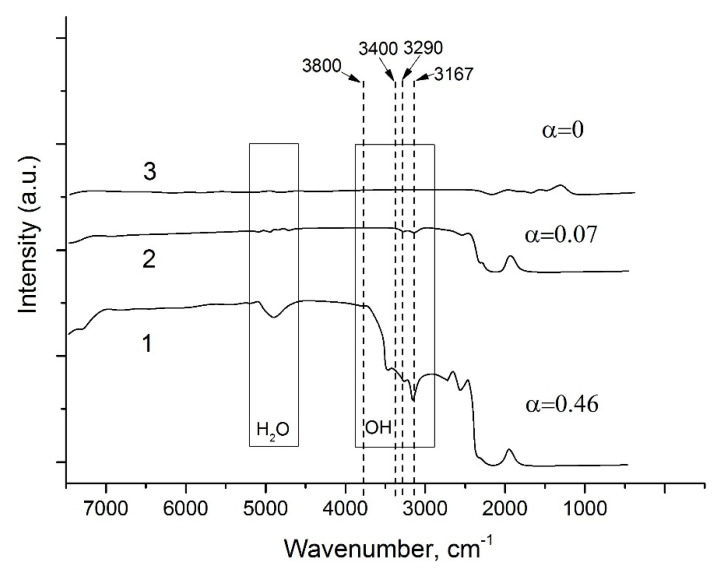
IR spectra of GaPO_4_ crystals obtained under follow conditions: 1—hydrothermal solution, 9 *M* H_3_PO_4_/12 *M* HCl (80/20), T = 240 °C, dT = 10 °C, refluxing method, isometric crystals; 2—hydrothermal solution, 6 *M* H_2_SO_4_, T = 230 °C, dT = 4 °C, temperature gradient method, plate-like crystals, 3—flux, lithium molybdate, T = 750–900 °C, decreasing of temperature, millimeter size crystals.

The first condition for precise determination of OH^−^ content is a high structural homogeneity of crystals, absence of cracks and gas–liquid inclusions (mother solution impurity-free). As grown by the refluxing method, GaPO_4_ crystals satisfied this condition. The refluxing method applied to the growth of GaPO_4_ crystals showed that the solvents and T–P conditions similar to hydrothermal slow temperature gradient method allowed obtaining GaPO_4_ single crystals characterized by relatively low OH^−^ content (Figure 9) (at 220–240 °C and pressure up to 20 bar). Alpha (**α**) value (parameter for characterization of OH^−^ content [34,35] of the grown GaPO_4_ single crystals by the refluxing method does not exceed 0.5. The **α** values of GaPO_4_ crystals grown by different methods are presented in Table 2 and Figure 9.

## 3. Materials and Methods

### 3.1. Main Features of the Hydrothermal Refluxing Method

The main features of the hydrothermal refluxing method consists of the following: a low filling of the autoclave with an initial solution (up to 50–60%); seeds and a nutrient material are placed in two principally different zones of the autoclave; seeds are found in a solution (in the bottom of the autoclave), but a nutrient is above the solution. Mass transport is carried out by evaporation of the solvent, condensation in the “cold” upper part of the autoclave (refrigerator) followed by diffusion into the nutrient basket, dissolution of the nutrient material and then, dropping down into the solution in the bottom part of the autoclave. As a result of the multiple recirculation (refluxing) of the solvent, the initial solution is gradually saturated and maintains this supersaturation for the full crystal growth cycle.

The practical application of the refluxing method for α-GeO_2_ crystal growth and the base of previous studies on GaPO_4_ crystal growth have driven us to experimentally test the refluxing method to grow GaPO_4_ crystals.

### 3.2. Experimental Procedure

**Figure 10:**
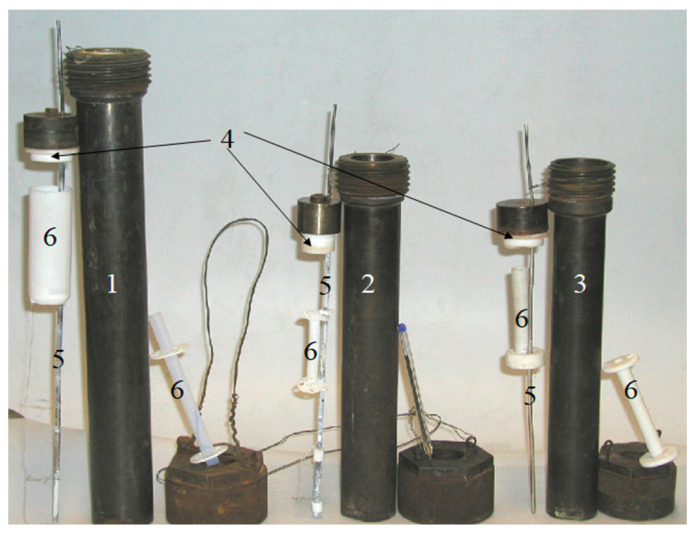
(1,2) Ta- and (3) Pt-lined steel autoclaves with PTFE cover; (4) autoclaves equipped with an (5) internal temperature control probe and (6) PTFE nutrient basket. Pen for scale.

For the first time, crystal growth vessels (autoclaves) were designed and approved for growing of GaPO_4_ by the refluxing method. A wide range of temperatures and solvents considered for the experiments led us to use platinum (Pt), tantalum (Ta) and polytetrafluoroethylene (PTFE) liners for the high pressure and temperature steel autoclaves. Precise temperature control was realized with the help of an internally inserted temperature control probe (thermocouple Type K). All autoclaves included a PTFE nutrient basket (Figure 10). Using the PTFE equipment prevented carrying out the experiments at temperatures above 250 °C. The estimated limiting factor for the hydrothermal refluxing method should be a critical temperature of a substance (the temperature at and above which vapor of the substance cannot be liquefied, no matter how much pressure). In our case, the limiting substances were aqueous solutions with well known critical temperature for water near 370 °C.

According to the known conditions of GaPO_4_ crystallization, in order to obtain the lowest OH^−^ content in the crystals, the most suitable solvents are aqueous H_3_PO_4_, H_2_SO_4_ and HCl solutions or their mixtures and temperatures exceeding 200 °C. These temperatures correspond to retrograde or constant solubility of GaPO_4_ (see Figure 1).

This is why we cannot use a unsaturated solution for starting crystal growth of GaPO_4_ crystals, as it risks dissolving seeds. In other words, the initial (starting) solution must be already saturated with GaPO_4_ at the precise concentration of GaPO_4_ corresponding to the equilibrium temperature to avoid spontaneous crystallization or dissolution of the seed.

**Figure 11:**
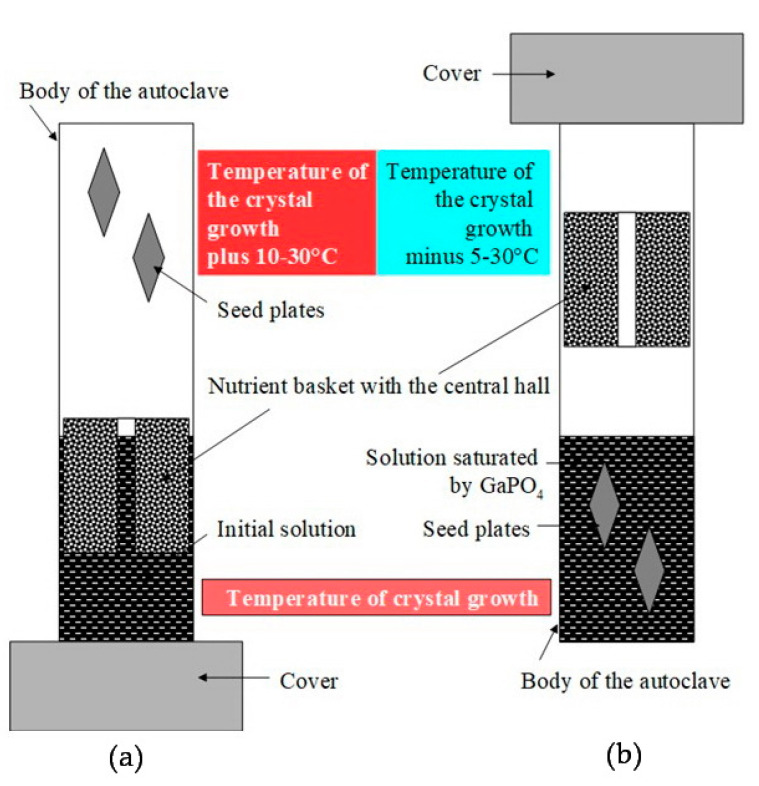
Runs sketch, saturation of the initial solution and further growth of GaPO_4_ crystals in the same run cycle. (**a**) First part of the run, autoclave is installed with the seeds on the top and nutrient– in the down; (**b**) second part of the run: autoclave is turned over, the seeds take a place in the saturated solution and the nutrient remains above.

**Figure 12:**
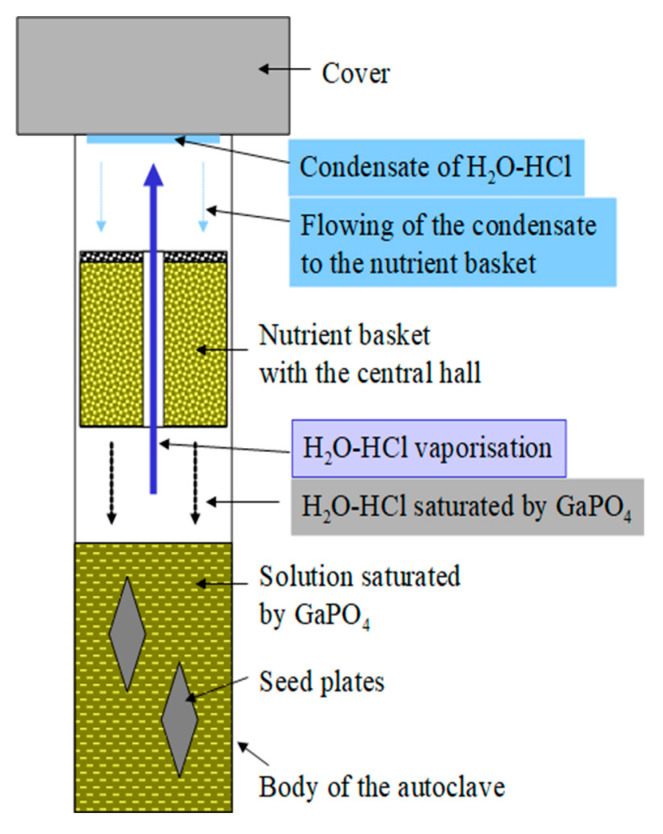
Principal sketch of the refluxing system in an autoclave. Mass transport in the refluxing system: a mother solution is in the bottom part of the autoclave, presented by “heavy” acids as the mineralizer, and a low-vapor-pressure acid is the conveyor of dissolved GaPO_4_ from nutrient to the mother solution.

Improvements of the refluxing method used for α-GeO_2_ crystal growth concerning conditions of GaPO_4_ crystal growth has allowed carrying out the saturation of initial solution and further growth of GaPO_4_ crystals in the same run cycle. This does not interrupt the course of the run, and as a consequence, neither spontaneous crystals nor losing of seed were observed. Technically, the following steps are in the first part of run: the autoclave is charged by solvent, nutrient and seeds are installed with the seeds on the top and nutrient below. The temperature is 10–30 °C higher in the top of autoclave (Figure 11a) than the bottom (to prevent evaporated solution convection). In the bottom, a working temperature of the future crystal growth is set. After completing the saturation of the solution by GaPO_4_, the autoclave is turned over. Thus, seeds take hold in the saturated solution (Figure 11b) at equilibrium temperature, and the nutrient remains above them. The difference in temperatures between the mother solution (bottom) and the refrigerator (top part of the autoclave) initiates the evaporation of the solvent and its condensation on the surface of the refrigerator. The process continues, as described before, as the condensate flows to the nutrient basket where the dissolution of GaPO_4_ nutrient occurs, followed by the dropping down of the saturated solution from the nutrient basket to the bottom part of the autoclave. The recirculation of the solvent in the given cycle results in a gradual super saturation of the initial solution. It maintains the saturation of the mother solution necessary for GaPO_4_ crystals growth at all times of the run. The principal schema of the refluxing system in an autoclave is shown in Figure 12.

**Figure 13:**
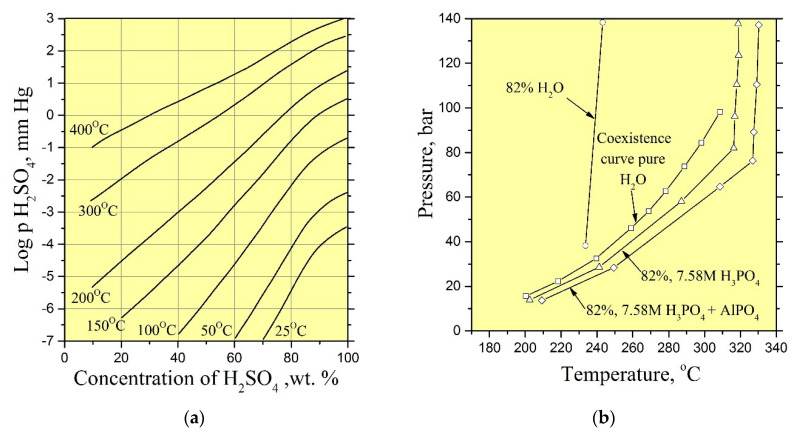
(**a**) Partial pressure of H_2_SO_4_ over aqueous sulfuric acid [37]; (**b**) pressure versus temperature at fill of 82% for H_2_O, 7.58 M H_3_PO_4_ and 7.58 M H_3_PO_4_ saturated with AlPO_4_ [38].

As reported by [10,18,29,31], good quality crystals of GaPO_4_ have been grown in aqueous solutions of (H_3_PO_4_ and H_2_SO_4_) acids at temperatures of 170–260 °C and pressures up to 50 bar. We used the same solutions as mineralizers in our experiments. However, the vapor pressure of such acids that excludes a formation of condensate in sufficient quantities at temperatures up to 250 °C is low (Figure 13a,b). Consequently, solutions of H_3_PO_4_ and H_2_SO_4_ could not be a solvent for the nutrient. The addition of low-vapor-pressure solvents, for example, hydrochloric acid (HCl) to the initial solution allowed increasing nutrient dissolution under vapor/condensate recirculation conditions.

In summary, the real process of mass transport could be represented as: a mother solution in the bottom part of the autoclave, represented by “heavy” (relatively low vapor-pressure) acids as the mineralizer and a low-vapor-pressure acid as the conveyor of dissolved GaPO_4_ from the nutrient to the mother solution (see Figure 12).

Plates (with sizes up to 45 × 15 × 1 mm) cut from GaPO_4_ crystals grown by slow temperature rise method in aqueous solution of 9 *M* H_3_PO_4_ at temperatures of 150–165 °C and pressure up to 5 bar were used as seeds. Different crystallographic orientations c{0001}, s{$11\bar{2}\;0$}, r{$10\bar{1}1$}, z{$01\bar{1}1$}, π{$10\bar{1\;}2$}, π’{$01\bar{1}2$} and x{$12\bar{1}0$} were cut.

The morphology and internal structure of the obtained GaPO_4_ crystals were studied on the surface and in polished cuts by optical microscopyMBS-9 (Russia), ADF STD16 (China), Nikon Eclipse LV100Pol (Japan). The IR spectroscopy of the crystals or polished plates was performed on PerkinElmer 820 spectrometer (USA). The OH^−^ content in crystals were estimate by α value, which was calculated using the formula *α* = (1/*l*)*(Log(T3800/T3400))-0,078, where: *l*—thickness of sample in cm, T3800 and T3400—intensity of absorption at wave numbers of 3800 and 3400 cm^−1^, 0.078—absorption of the intrinsic lattice vibrations of GaPO_4_ at 3400 cm^−1^ [34,35]. The presence of defects in the crystal plates was identify by X-ray topography at IMPMC UMR CNRS 7590 (Montpellier, France) by a homemade device using a Cu Kα tube.

## 4. Conclusions

The GaPO_4_ crystals grown by the hydrothermal refluxing method at temperatures close to 240 °C and pressures up to 10–20 bar in aqueous solutions of the H_3_PO_4_–HCl mixtures have a relatively low OH^−^ content and considerably high structural uniformity in comparison to crystals grown by other low temperature (< 250 °C) hydrothermal methods.

Hydrothermal refluxing method of GaPO_4_ crystals growth has shown a very high reproducibility of the results.

Improvement of the hydrothermal refluxing method concerning application to GaPO_4_ crystal growth gave a new technique of measurement of GaPO_4_ concentration in solution at set parameters. The presented technique allows increasing the accuracy of GaPO_4_ concentration definition in aqueous solutions of H_3_PO_4_, H_2_SO_4_, HCl and their mixtures at least up to 250 °C and pressures of 10–30 bar.

## Figures and Tables

**Table 1:** Main results of GaPO_4_ crystal growth runs by the hydrothermal refluxing method.

N	Solution Composition, Vol. Proportion	Crystallization Temperature, °C	Gradient dT, °C	Crystal Growth Rates, mm/day		IR, α	Size of Grown Crystals, Y–X–Z mm
				Vx	Vz		
1	9 M H_3_PO_4_/12 M HCl, 90/10	180	20	0.44	>0.61 *	5	26.8–16.5–4.2
2	9 M H_3_PO_4_/12 M HCl, 95/5	220	20	0.28	>>0.37 *	0.8	47.5–11.3–5.7
3	9 M H_3_PO_4_/12 M HCl, 95/5	226	20	0.23	>0.44 *	0.5	33.8–18.0–7.9
4	9 M H_3_PO_4_/12 M HCl, 95/5	236	16	0.21	>>0.12 *	0.4	49.7–19.7–11.3
5	9 M H_3_PO_4_/12 M HCl, 95/5	240	25	0.18	>>0.18 *	0.4	47.9–19.3–7.6
6	5 M H_3_PO_4_/12 M HCl, 97.5/2.5	220	10	0.03	>0.04	–	30.0–18.5–1.34
7	5 M H_3_PO_4_/12 M HCl, 95/5	220	20	1.05	>0.86 *	10	16.6–11.2–8.3
8	5 M H_3_PO_4_/12 M HCl, 95/5	230	15	0.09	0.13	0.4	14.5–10.2–4.4
9	6 M H_2_SO_4_/12 M HCl, 95/5	220	20	0.001	0.001	–	–
10	9 M H_3_PO_4_	220	20	0	0	–	–
11	6 M H_2_SO_4_	225	20	0	0	–	–

*—solution—the top part of the autoclave—and the nutrient basket, in other words, the temperature gradient.

**Table 2:** Comparative alpha values of GaPO_4_ crystals grown by temperature gradient, slow temperature rise, refluxing hydrothermal methods and by flux method.

Method	Crystal Growth Temperature, °C	Solution	Crystal Growth Rates, mm/day		IR, α	Reproducibility (In Situ)
			Vx	Vz		
Slow temperature rise	150–185	9 *M* H_3_PO_4_	0.5–0.8	0.1–0.2	1.0–1.5	+
Temperature gradient	176	9 *M* H_3_PO_4_/6 *M* H_2_SO_4_, 20/80	0.5–0.8	0.05–0.1	0.5–1.0	+/−
Temperature gradient	230	6 *M* H_2_SO_4_	0.8–1.5	0.01–0.05	0.04–0.1	+/−
Refluxing	230	9 *M* H_3_PO_4_/12 *M* HCl, 95/5	0.1–1.2	0.1–1.0	<0.5	+
Flux	900–750	Lithium molybdate	0.25	0.05	0	+/−

## In Memory of Professor Nikolay Leonyuk

For all of us he will be missed. Inspired by his modesty, determination and honesty we will remember him as a great crystal grower, educator and a true gentleman.
